# Novel galeterone analogs act independently of AR and AR-V7 for the activation of the unfolded protein response and induction of apoptosis in the CWR22Rv1 prostate cancer cell model

**DOI:** 10.18632/oncotarget.19762

**Published:** 2017-08-01

**Authors:** David J. McCarty, Weiliang Huang, Maureen A. Kane, Puranik Purushottamachar, Lalji K. Gediya, Vincent C.O. Njar

**Affiliations:** ^1^ Department of Pharmacology, University of Maryland School of Medicine, Baltimore, MD 21201, USA; ^2^ Center for Biomolecular Therapeutics, University of Maryland School of Medicine, Baltimore, MD 21201, USA; ^3^ Department of Pharmaceutical Sciences, University of Maryland School of Pharmacy, Baltimore, MD 21201, USA; ^4^ Marlene and Stewart Greenebaum Comprehensive Cancer Center, University of Maryland School of Medicine, Baltimore, MD 21201, USA

**Keywords:** CWR22Rv1, apoptosis, AR-V7, prostate cancer, unfolded protein response

## Abstract

The androgen receptor (AR) has long been the primary target for the treatment of prostate cancer (PC). Despite continuous efforts to block AR activity through ligand depletion, AR antagonism, AR depletion and combinations thereof, advanced PC tumors remain resilient. Herein, we evaluate two galeterone analogs, VNPT-178 and VNLG-74A, in PC cell models of diverse androgen and AR dependence attempting to delineate their mechanisms of action and potential clinical utility.

Employing basic biochemical techniques, we determined that both analogs have improved antiproliferative and anti-AR activities compared to FDA-approved abiraterone and enzalutamide. However, induction of apoptosis in these models is independent of the AR and its truncated variant, AR-V7, and instead likely results from sustained endoplasmic reticulum stress and deregulated calcium homeostasis. Using in silico molecular docking, we predict VNPT-178 and VNLG-74A bind the ATPase domain of BiP/Grp78 and Hsp70-1A with greater affinity than the AR.

Disruption of 70 kDa heat shock protein function may be the underlying mechanism of action for these galeterone analogs. Therefore, despite simultaneously antagonizing AR activity, AR and/or AR-V7 expression alone may inadequately predict a patient’s response to treatment with VNPT-178 or VNLG-74A. Future studies evaluating the context-specific limitations of these compounds may provide clarity for their clinical application.

## INTRODUCTION

Prostate cancer (PC) is among the most frequently diagnosed and lethal malignancies in American men [1]. While increased early detection combined with surgical or radiological treatment of localized tumors has significantly improved the overall survival rate, nearly all men treated with systemic therapies (*e.g.* castration) targeting advanced or metastatic tumors develop resistance. New approaches are therefore necessary to combat the progression of these lesions to the lethal phenotype referred to as castration-resistant prostate cancer (CRPC).

The androgen receptor (AR) is a transcription factor belonging to the nuclear hormone family of receptors and continues as the primary target for PC drug development as its role in the maintenance of PC tumors in all stages of progression is well-documented [2]. AR splice-variants (AR-Vs) are C-terminally truncated proteins lacking part or all of the ligand-binding domain (LBD) and are therefore unaffected by drugs promoting the depletion of androgens or competitive antagonism of the AR. Abiraterone acetate (Zytiga) and enzalutamide (Xtandi), the two most-recent AR-directed therapies to gain FDA approval for treatment of PC, are completely ineffective in men presenting with AR-V7-expressing tumors [3].

Consequently, many research efforts have focused on the evaluation of natural and synthetic small molecules capable of promoting the depletion of the AR protein, including AR-Vs, in addition to disruption of its activity. Although some of these AR depleting agents (ARDAs) have demonstrated promising results in preclinical models of CRPC, the Phase 3 clinical failure of galeterone versus enzalutamide [NCT02438007] reinforces our need for improved understanding of their mechanisms of action as well as potential context-specific limitations of their use [4, 5]. Indeed, while the ubiquitin-proteasome pathway is largely responsible for the degradation of the AR under normal conditions [6], the specifics underlying its enhanced selection of the AR and AR-Vs following ARDA treatments are incompletely delineated.

In eukaryotes, the 26S proteasome functions as the primary machinery for the recycling of soluble proteins but it requires that these targets be specifically marked by K48-linked polyubiquitination; ergo, the ubiquitin-proteasome pathway is largely selective of proteins that are in excess, misfolded, or generally nonfunctioning. It has been shown that galeterone is capable of directly binding the AR-LBD and comparative studies have indicated treatments of galeterone and the proteasome inhibitor MG132 both promote the accumulation of polyubiquitinated AR proteins [7, 8]. However, these observations are not unique as inhibitors of Hsp90, a ubiquitous chaperone protein, also promote AR polyubiquitination and degradation indicating potential for altering the steady-state expression of the AR without directly binding the receptor itself [9].

Early studies of galeterone action in AR-negative PC cell lines also revealed its ability to activate the unfolded protein response (UPR)/endoplasmic reticulum stress response (ERSR) [10]. The endoplasmic reticulum (ER) is heavily relied upon by epithelial cells for the maintenance of protein homeostasis as secretory and membrane proteins require the ER and trans-Golgi network for proper processing and trafficking to the plasma membrane. During times of cellular stress, an abundance of misfolded proteins can accumulate in the ER lumen triggering the UPR which functions largely as a survival pathway by attenuating global protein synthesis and increasing the cell’s capacity for chaperone-mediated protein refolding or clearance. Activation of the UPR can therefore result in increased proteasome activity and autophagy to restore homeostasis; however, if a sustained UPR occurs at high levels, cells may instead undergo apoptotic death [11].

While previous galeterone studies demonstrated its activation of the UPR and the proteasome-mediated depletion of the AR/AR-V7, no additional targets have been identified that could help to explain these separate biological responses. We believe these phenomena are linked, if not by a common mediator, by a motif or domain shared by distinct proteins with critical roles in maintaining AR stability and ER homeostasis. Therefore, herein we evaluate the biological activities of two galeterone analogs that have demonstrated improved potential in our preliminary studies. Using basic biochemical assays and optimized experimental models, we conclude that VNPT-178 and VNLG-74A antagonize the full-length AR (fAR), promote its post-translational depletion and initiate and sustain activation of the UPR leading to induction of apoptosis. In addition to our functional analyses, in silico molecular docking reveals the 70 kDa heat shock protein chaperones BiP/Grp78 and Hsp70-1A may be primary targets for VNPT-178 and VNLG-74A. To our knowledge, these data are the first to reconcile two of the divergent cellular responses to ARDA treatments and may provide additional insight to potential advantages and caveats associated with studies of galeterone analogs.

## RESULTS AND DISCUSSION

### VNPT-178 and VNLG-74A impair the proliferation of PC cells *in vitro*

**Figure 1:**
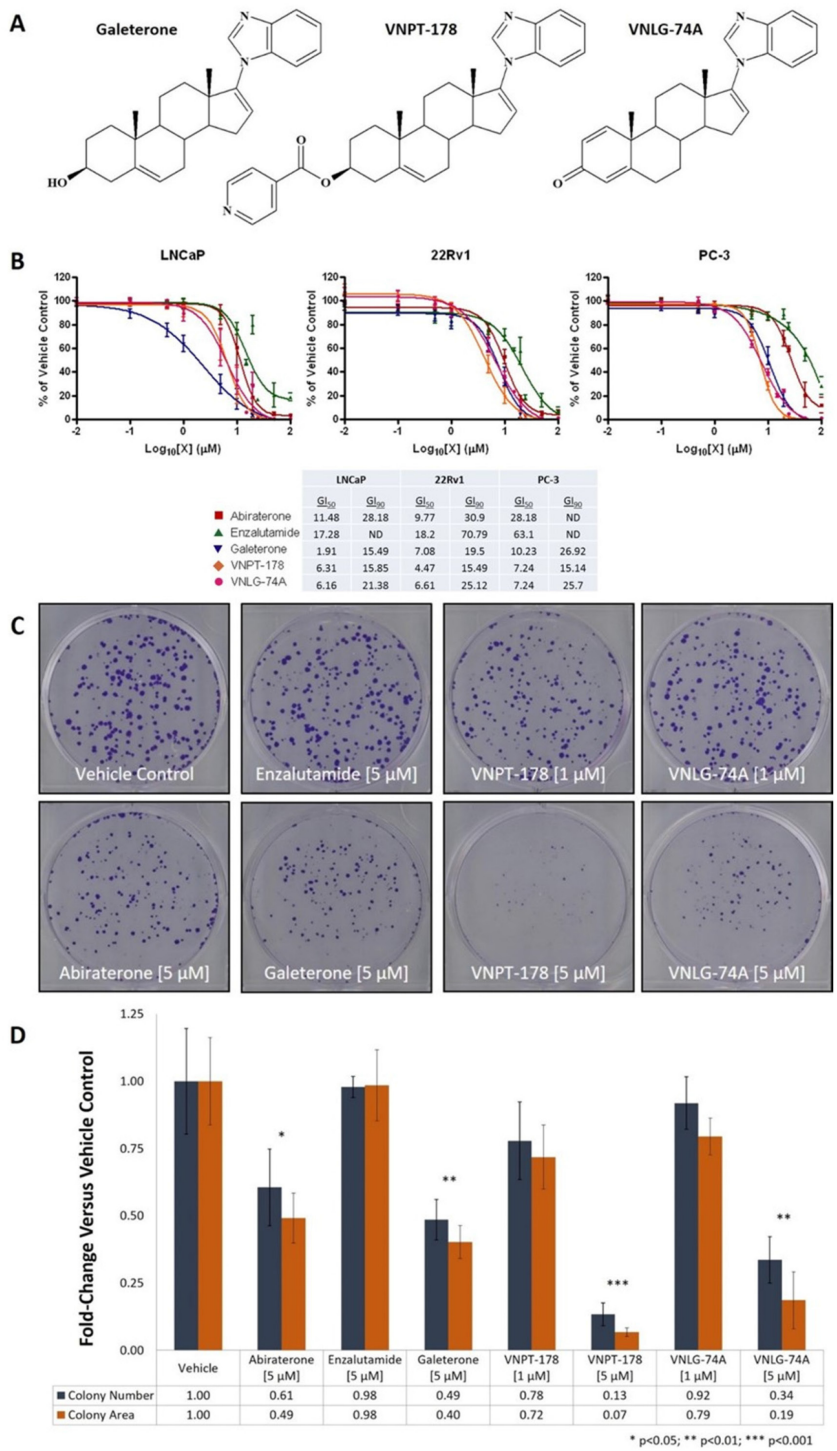
Galeterone analogs VNPT-178 and VNLG-74A inhibit the proliferation of PC cell lines *in vitro* **(A)** Structures of galeterone, VNPT-178, and VNLG-74A. **(B)** MTT viability analysis of the antiproliferative actions of VNPT-178 and VNLG-74A compared to abiraterone, enzalutamide, and galeterone in LNCaP, 22Rv1, and PC-3 cells. Plots represent mean ± SEM of at least three independent experiments and the estimated GI_50_ and GI_90_ values are summarized in the table below. **(C-D)** 22Rv1 colony formation is inhibited by 5 μM treatments of both VNPT-178 and VNLG-74A. Colonies were stained with crystal violet (C) and the mean number and area of the colonies within three independent treatment groups were compared to those of vehicle controls (D).

Our first objective was to evaluate the antiproliferative activity of galeterone analogs VNPT-178 and VNLG-74A (Figure 1A) in widely-used PC cell models of diverse androgen and AR dependence. LNCaP cells predominantly express a T878A mutated fAR and are sensitive to and largely dependent on androgen signaling for survival. CWR22Rv1 (22Rv1) cells express an H875Y mutated fAR as well as the truncated, constitutively-active variant AR-V7 and therefore represent a cell model of androgen sensitivity and androgen independence. Alternatively, PC-3 cells do not express the AR and are insensitive to and independent of androgens for growth and viability. Using an MTT assay to measure dose-dependent changes in viable cell populations after six days of treatment, VNPT-178 and VNLG-74A demonstrated improved potencies compared to abiraterone and enzalutamide (Figure 1B *plots*) with 50 and 90% growth inhibitory (GI_50_ and GI_90_) concentrations estimated in the low micromolar range (Figure 1B *table summary*). As with galeterone, both VNPT-178 and VNLG-74A elicit similar responses in all three cell lines while the AR-negative PC-3 cells are markedly resistant to abiraterone and enzalutamide.

The AR is reported to function as a regulator of DNA replication and cell cycle progression in PC cells and therefore longer time points may enhance the relative efficacy of ARDAs administered at concentrations below our estimated GI_50_ values [12]. 22Rv1 cells were seeded at low density and adherent cell colonization was monitored after 15 days in the presence of vehicle, abiraterone, enzalutamide, or ARDA (Figures 1C-1D). Compared to the MTT results, increasing treatment time did improve the relative antiproliferative effect of abiraterone and the ARDAs while an equimolar concentration of enzalutamide was insufficient to impair 22Rv1 colony development.

### VNPT-178 and VNLG-74A antagonize the fAR

**Figure 2:**
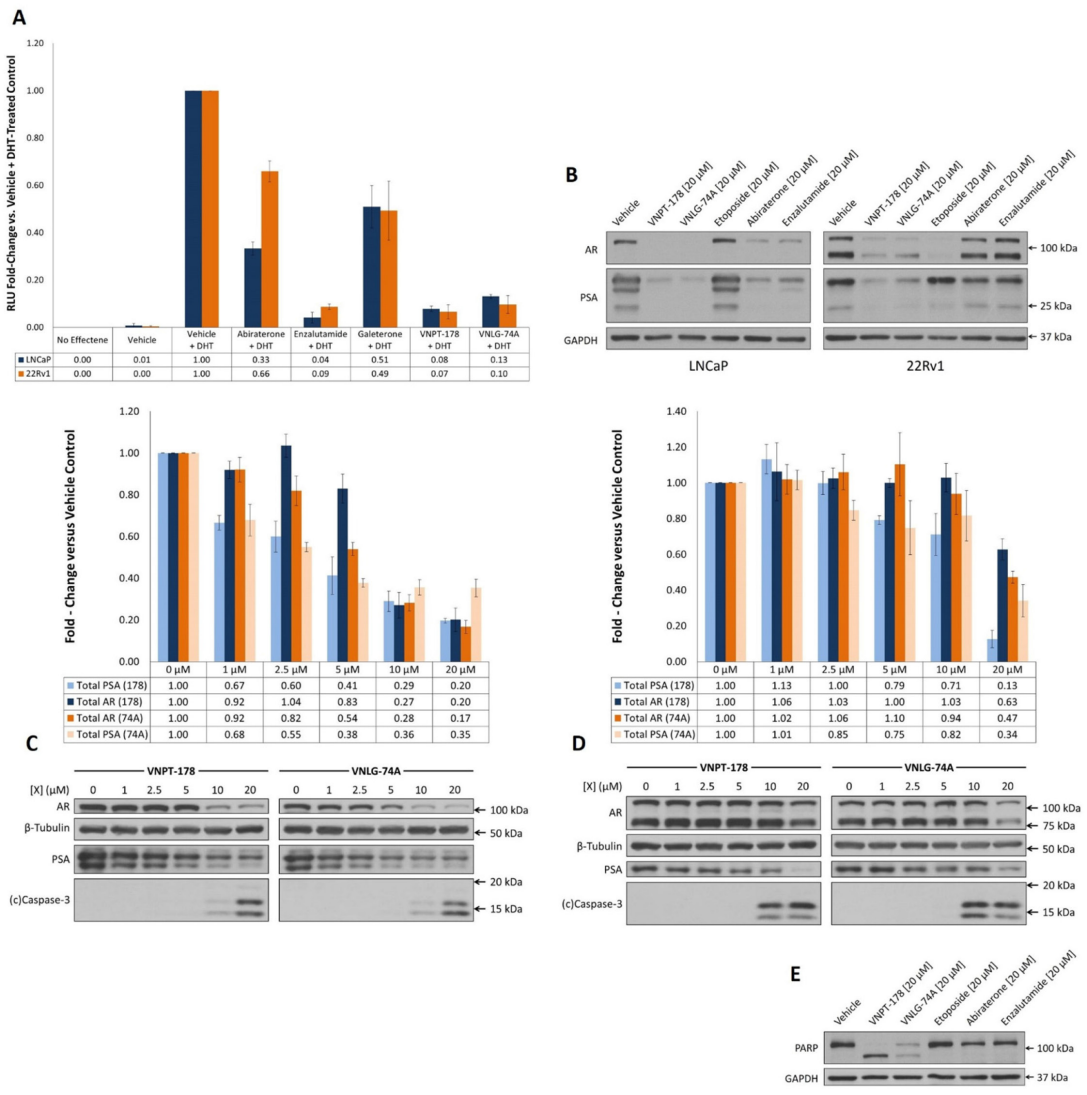
VNPT-178 and VNLG-74A antagonize the fAR and promote its depletion **(A)** Androgen-dependent luciferase expression was determined in LNCaP and 22Rv1 cells transiently co-transfected with ARR2-Luc and pRL-null reporters. Plot represents mean RLU fold-change ± SEM of three independent experiments. **(B)** Western blots of AR and PSA expression in LNCaP and 22Rv1 cells 48 hours after treatment with indicated compounds. **(C)** LNCaP and **(D)** 22Rv1 cells treated with increasing doses of VNPT-178 or VNLG-74A for 24 hours exhibit differences in AR, PSA, and cleaved caspase-3 expression. Densitometry of total AR and PSA immunoblots are plotted as the mean fold-change ± SEM of three replicate assays. **(E)** VNPT-178 and VNLG-74A uniquely promote PARP cleavage in LNCaP cells after 48 hours.

Since the AR remains a primary target for PC drug development we next sought to determine whether VNPT-178 or VNLG-74A could antagonize its transcriptional activation. LNCaP and 22Rv1 cells were cultured in media supplemented with charcoal/dextran-stripped FBS for 24 hours to deplete residual androgens before co-transfection with firefly (ARR2-Luc) and Renilla (pRL-null) luciferase reporter constructs. Following a four-hour pre-incubation with 10 μM of each compound or vehicle, 10 nM dihydrotestosterone (DHT) was added and the ratio of luciferase activity was measured 20 hours later (Figure 2A). Without DHT, virtually zero firefly luciferase activity was detected following substrate addition in both LNCaP and 22Rv1 cells supporting the ARR2-modified rat Probasin promoter is highly androgen-sensitive and poorly targeted by AR-V7 [13]. Here, equimolar concentrations of enzalutamide, VNPT-178, and VNLG-74A are more effective than galeterone or abiraterone by inhibiting approximately 90% of the transcriptional activation of fAR in both cell lines.

### VNPT-178 and VNLG-74A reduce total AR and PSA protein expression in LNCaP and 22Rv1 cells

In accordance with other studies [14], we have demonstrated that full-length AR antagonism alone is insufficient to inhibit the proliferation of 22Rv1 cells/colonies. Therefore, unlike enzalutamide, both VNPT-178 and VNLG-74A must either possess some activity against AR-V7 or are able to inhibit 22Rv1 cell growth despite expression of AR-V7. Using SDS-PAGE and western blotting, we next assessed whether VNPT-178 and VNLG-74A treatments had an appreciable impact on the endogenous protein expression of AR and PSA (regulated by both fAR and AR-V7) in LNCaP and 22Rv1 cells. Both compounds were compared against equimolar concentrations of established agents or vehicle control and, after 48 hours, whole-cell lysates were collected and analyzed (Figure 2B). Consistent with reports on galeterone, both VNPT-178 and VNLG-74A exhibit improved AR-depletive and AR-antagonistic activity compared to abiraterone and enzalutamide [15, 16].

While all four AR-directed compounds effectively reduced AR and PSA expression in LNCaP cells, antagonism of the fAR is likely commensurate with increased AR-V7 activity in 22Rv1 cells. Indeed, abiraterone and enzalutamide have minimal impact on endogenous AR and PSA expression in 22Rv1 cells while both VNPT-178 and VNLG-74A retain their activity. Unexpectedly, the topoisomerase-II inhibitor etoposide profoundly reduced total AR expression in 22Rv1 cells with minimal change of PSA levels. As this effect was not observed in LNCaP cells, it is difficult from these data to ascertain a specific mechanism though it may be related to retention of PSA in Golgi vesicles during a DNA damage response [17].

Higher concentrations (>10 μM) of VNPT-178 and VNLG-74A have routinely caused extensive cell death in both LNCaP and 22Rv1 cells (*data not shown*). Therefore, to identify possible dose-dependent activities, both cell lines were treated with increasing concentrations of VNPT-178 and VNLG-74A and the expression of AR, PSA, and cleaved caspase-3 was evaluated by western blot (Figures 2C-2D). With only the fAR present, increasing the ARDA concentration in LNCaP cells results in a predictable pattern of decreased AR and PSA expression with concomitant increases in caspase-3 cleavage by 24 hours (Figure 2C). 22Rv1 cells, on the other hand, do not exhibit the same response as these data show that while at least 20 μM ARDA or additional time is necessary to substantially deplete AR expression and activity, 10 μM is sufficient to induce equal caspase-3 activation (Figure 2D).

As we were able to see substantial AR and PSA depletion in LNCaP cells treated with ARDA, abiraterone, and enzalutamide for 48 hours (Figure 2B *left panel*), we took a second look at the possibility of concomitant cell death and found, unexpectedly, that only VNPT-178 and VNLG-74A promoted appreciable PARP cleavage (Figure 2E). Taken together, these data raise the question as to whether ARDA-induced cell death within 48 hours is in fact a result of potent AR antagonism/depletion or if another mechanism is responsible.

### Incomplete depletion of fAR and/or AR-V7 is insufficient to reduce PSA expression or induce apoptosis in 22Rv1 cells

**Figure 3:**
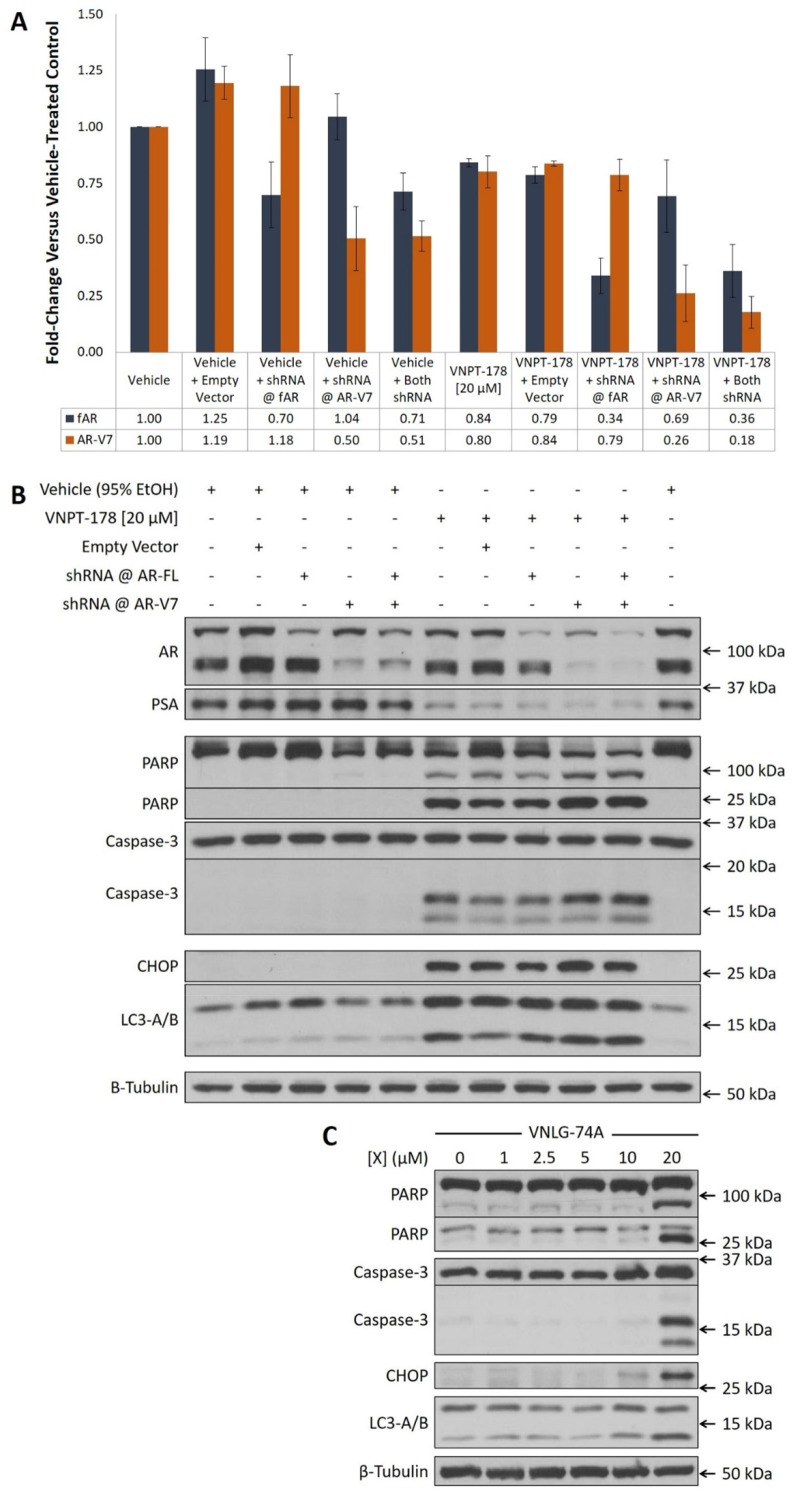
VNPT-178 and VNLG-74A induce apoptosis independently of the AR **(A)** shRNA against fAR and AR-V7 selectively reduce AR expression at 72 hours in 22Rv1 cells. Immunoblot densitometry plots of mean fold-change ± SEM. **(B)** Western blots showing 72-hour AR depletion is insufficient to induce apoptosis in 22Rv1 cells compared to the addition of 20 μM VNPT-178 for 24 hours. **(C)** 20 μM VNLG-74A can induce apoptosis in PC-3 cells at 96 hours.

The AR is highly expressed in many PC cells (and tumors) and therefore it is likely to be overly abundant relative to a necessary threshold to maintain their growth [18]. To test this hypothesis, we performed selective shRNA knockdown of fAR and/or AR-V7 in 22Rv1 cells and evaluated its effect on PSA expression and apoptosis induction after 72 hours. As can be seen in Figures 3A-3B, while each shRNA specifically reduced abundance of its target AR isoform, neither their individual nor combined administration markedly reduced PSA expression. Similarly, neither an estimated 30% reduction in fAR nor 50% reduction in AR-V7 (individually or combined) was sufficient to induce marked caspase-3 or PARP cleavage. On the contrary, the addition of a 24-hour treatment of VNPT-178 reduced PSA expression and promoted caspase-3 and PARP cleavage across all conditions (Figure 3B). While it may be argued that combining ARDA and shRNA treatments enhanced the depletion of AR protein below a threshold requirement and therefore caused these observations, when we look beyond the AR axis we can see that CHOP expression and LC3-lipidation is increased exclusively in the ARDA-treated conditions. Additionally, PC-3 cells respond similarly to ARDA (albeit at 96 hours instead of 24) with the upregulation of CHOP and LC3-lipidation and the cleavage of caspase-3 and PARP (Figure 3C) reinforcing the likelihood of an AR-independent response.

### ARDAs activate the UPR

**Figure 4:**
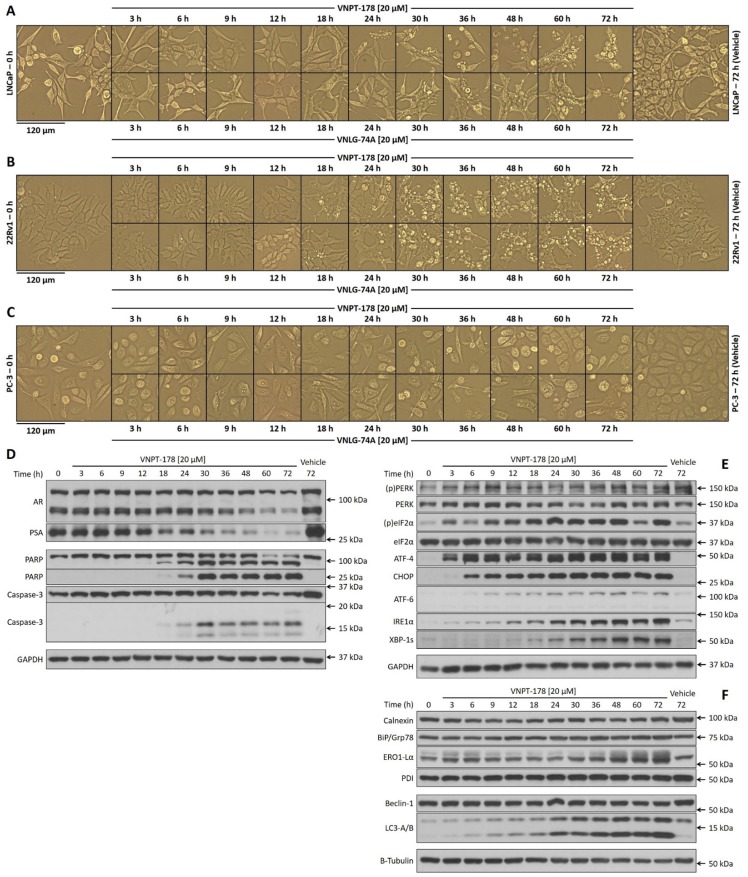
ARDAs rapidly initiate and sustain activation of the UPR Micrographs of LNCaP **(A)**, 22Rv1 **(B)**, and PC-3 **(C)** cells reveal morphological features of apoptosis following treatments with 20 μM VNPT-178 (*top panels*) and VNLG-74A (*bottom panels*) through 72 hours. Western blotting for markers of AR signaling and apoptosis **(D)**, UPR activation **(E)**, and ER chaperones and autophagy **(F)** reveal the correlation of AR antagonism to apoptosis is preceded by the UPR.

Prior studies with galeterone in PC-3 cells demonstrated its ability to rapidly promote ER calcium efflux to the cytosol—an effect that could be sustained for at least three hours [10]. Although the mechanisms were not elucidated, an activation of the UPR was identified. Our preliminary assessments at 24 hours revealed the UPR was activated by galeterone analogs and thapsigargin, but not by enzalutamide in LNCaP and 22Rv1 cells (*data not shown*). We were therefore interested in expanding our observations across multiple time points and evaluated LNCaP, 22Rv1, and PC-3 cellular responses to 20 μM VNPT-178 and VNLG-74A through 72 hours. Before sample collection for biochemical analysis, we collected micrographs of all three cell lines for each treatment condition and assembled a timeline for visual comparison (Figures 4A-4C). In LNCaP cells (Figure 4A), VNPT-178 (*top panels*) and VNLG-74A (*bottom panels*) treatments began to induce membrane blebbing, cell rounding, and cell detachment at approximately 24 hours and increased in proportions through 72 hours. 22Rv1 cells appear more sensitive to apoptosis induction with morphological characteristics appearing slightly ahead of LNCaP cells (∼18 hours; Figure 4B), while PC-3 cells appear resistant with less-pronounced morphological changes (Figure 4C).

As the 22Rv1 cells have exhibited peculiar responses to VNPT-178 and VNLG-74A already, it was of particular interest to evaluate the kinetics of AR and PSA expression relative to caspase-3 and PARP cleavage over a broader time frame. In Figure 4D, a modest decrease in PSA expression is first observed at 18 hours and is enhanced up to 72 hours correlating with an increase in caspase-3 and PARP cleavage and morphological apoptosis. Although the expression of both full-length and truncated AR proteins also diminishes over time, as seen previously, it is not completely depleted with ARDA treatment alone in 22Rv1 cells.

Next, we compared the expression of UPR effectors in 22Rv1 cells treated with ARDA. Within the first three hours, PERK and eIF2α phosphorylation as well as ATF4 expression are all observed, CHOP expression is seen by six hours, and spliced XBP-1 appears within 18 hours (Figure 4E) indicating the UPR is wholly engaged well ahead of PSA depletion or activation of apoptosis. Interestingly, IRE1α and ATF-6 which act as UPR sentinels are also steadily upregulated through 72 hours. Since it is the dissociation of BiP/Grp78 from these ER transmembrane proteins (as well as PERK) that is believed to activate the UPR in the first place, we next evaluated the expression levels of ER chaperones and found these proteins to be stably abundant (Figure 4F) [19]. Calnexin, BiP/Grp78, and PDI protein levels remain constant in 22Rv1 cells although an increase in ERO1-Lα is discernible. Studies have proposed IRE1α as indispensable for the upregulation of ER quality control and autophagy and the concomitant increase in LC3-lipidation with IRE1α expression may indicate the severity of an ARDA-induced UPR [20].

### Inhibition of caspases or calpains reverses ARDA-mediated PARP cleavage in 22Rv1 cells

Although we have reliably seen PARP cleavage in 22Rv1 cells following ARDA treatment and CHOP is a putative pro-apoptotic transcription factor, the UPR may not be exclusively responsible for the induction of cellular death seen here [21]. As stated, galeterone treatments in PC-3 cells promoted ER calcium efflux to the cytosol and while neither the mechanisms nor conductance channels were identified, sustained increases in cytosolic Ca^2+^ can itself promote apoptosis through several mechanisms including IP_3_ receptor activation, mitochondrial outer membrane permeabilization (MOMP) and release of cytochrome-c, and activation of p53 [22].

**Figure 5:**
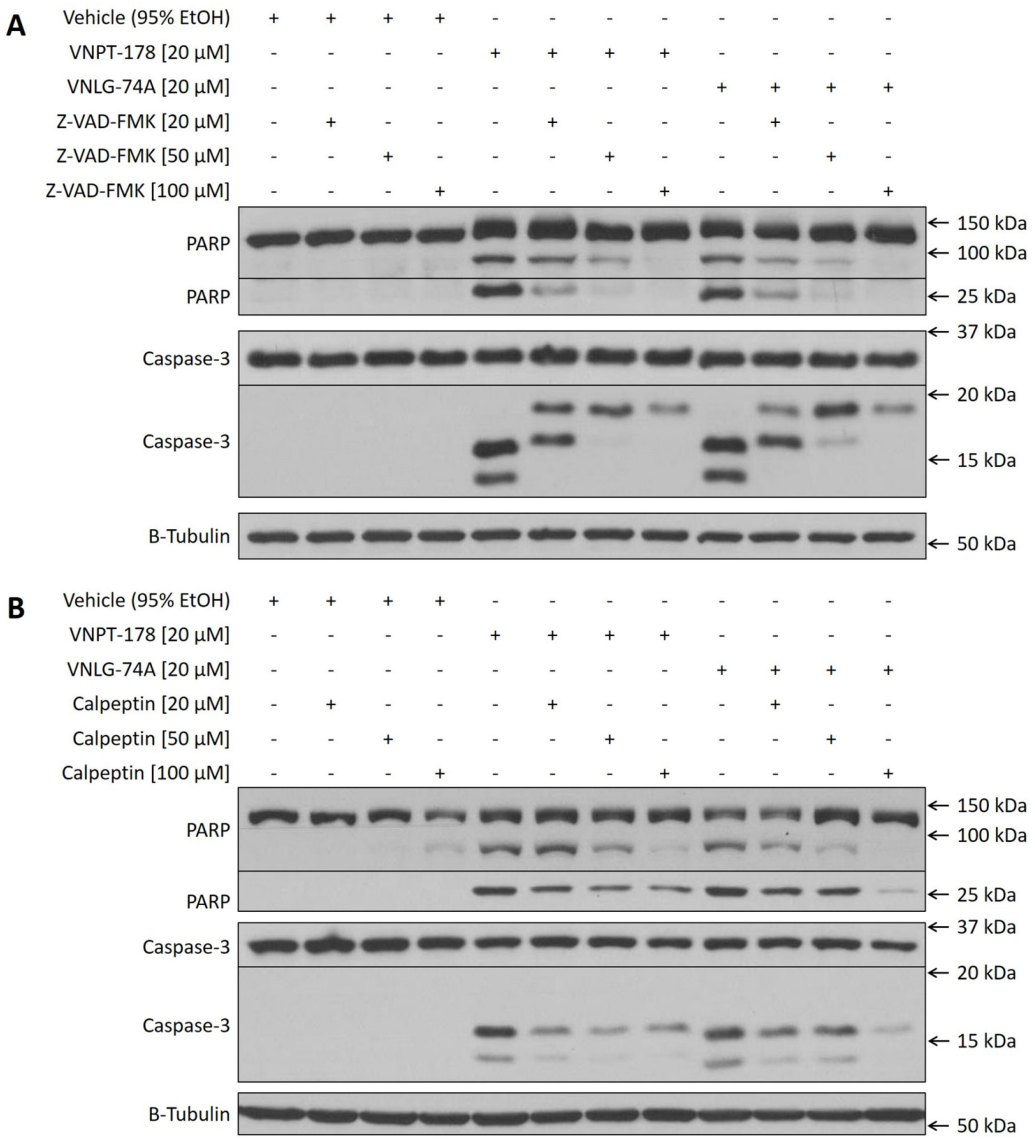
Caspase and calpain inhibition attenuates PARP cleavage following ARDA administration in 22Rv1 cells Whole-cell lysates of 22Rv1 cells treated with vehicle, VNPT-178, or VNLG-74A in the absence or presence of increasing doses of the pan-caspase inhibitor Z-VAD-FMK **(A)** or the calpain 1 and 2 inhibitor calpeptin **(B)** were collected for western blot analysis. Blots are representative of at least three independent experiments.

Therefore, we next sought to work backwards from PARP cleavage to identify a potential for Ca^2+^ to promote apoptosis. 22Rv1 cells were exposed to vehicle, VNPT-178 or VNLG-74A in the absence or presence of increasing concentrations of the pan-caspase inhibitor Z-VAD-FMK and whole-cell lysates were collected after 26 hours for analysis via western blot (Figure 5A). Z-VAD-FMK is able to block the catalytic activity of initiator and effector caspases and, expectedly, PARP cleavage following treatment with VNPT-178 and VNLG-74A is decreased in a dose-dependent manner corresponding to increased concentrations of Z-VAD-FMK. Unexpectedly, caspase-3 exhibited peculiar fragmentation in response to the co-treatment in 22Rv1 cells.

Caspase-3 is an aspartyl peptidase that exists natively as a 32 kDa zymogen and cleavage by an initiator peptidase yields two complementary fragments measuring 19/20 kDa and 12 kDa. The 19/20 kDa fragment of cleaved caspase-3 subsequently undergoes auto-proteolytic processing to produce a 17 kDa protein that forms heterotetrameric complexes with the 12 kDa fragment. While we expected to see an overall, dose-dependent decrease in cleaved caspase-3 proteins corresponding to increased inhibition of upstream caspases, we instead found a partially maintained abundance of 19/20 kDa caspase-3 fragments. Although 100 μM Z-VAD-FMK may be insufficient for a complete reversal of caspase-3 cleavage, it is possible that a non-caspase protease is promoting its activation here.

Calpains are calcium-dependent cysteine proteases with known roles in apoptosis [23]. Unlike caspases, calpain cleavage specificity is broader and likely determined by tertiary conformations versus primary sequence [24]. The calpain inhibitor calpeptin has been reported to attenuate apoptosis suggesting active calpains may modulate caspase-3 activation and, here, could sensitize 22Rv1 cells to apoptosis induction following ARDA treatments [25]. Similar to the Z-VAD-FMK inhibition, 20-100 μM treatments of calpeptin reduced caspase-3 and PARP cleavage in response to ARDA in 22Rv1 cells (Figure 5B). Relief of caspase inhibition reveals the typical pattern of caspase-3 cleavage fragments (17 and 12 kDa) and together these data suggest that calpains may be activated by ARDA-induced ER Ca^2+^ efflux, enhancing the apoptotic effects seen in 22Rv1 cells.

### VNPT-178 and VNLG-74A can interact with the ATPase domain of Hsp70-1A and BiP/Grp78

Taken together, our data suggest the galeterone analogs VNPT-178 and VNLG-74A, while able to antagonize the fAR, likely act independently of the AR axis to promote apoptosis through the release of Ca^2+^ from the ER and activation of the UPR. Due to their hydrophobic structures, these ARDAs could interact transiently with solvent-exposed residues on any number of proteins, particularly those localized to phospholipid membranes, but the consistency of their action suggests a strong preference for a specific target or class of targets. As the AR-LBD contains a large, hydrophobic binding cleft and the UPR is an early event following ARDA treatment, we focused our attention on BiP/Grp78 which contains a hydrophobic nucleotide-binding domain (ATPase domain) and rests at the center of UPR activation.

**Figure 6:**
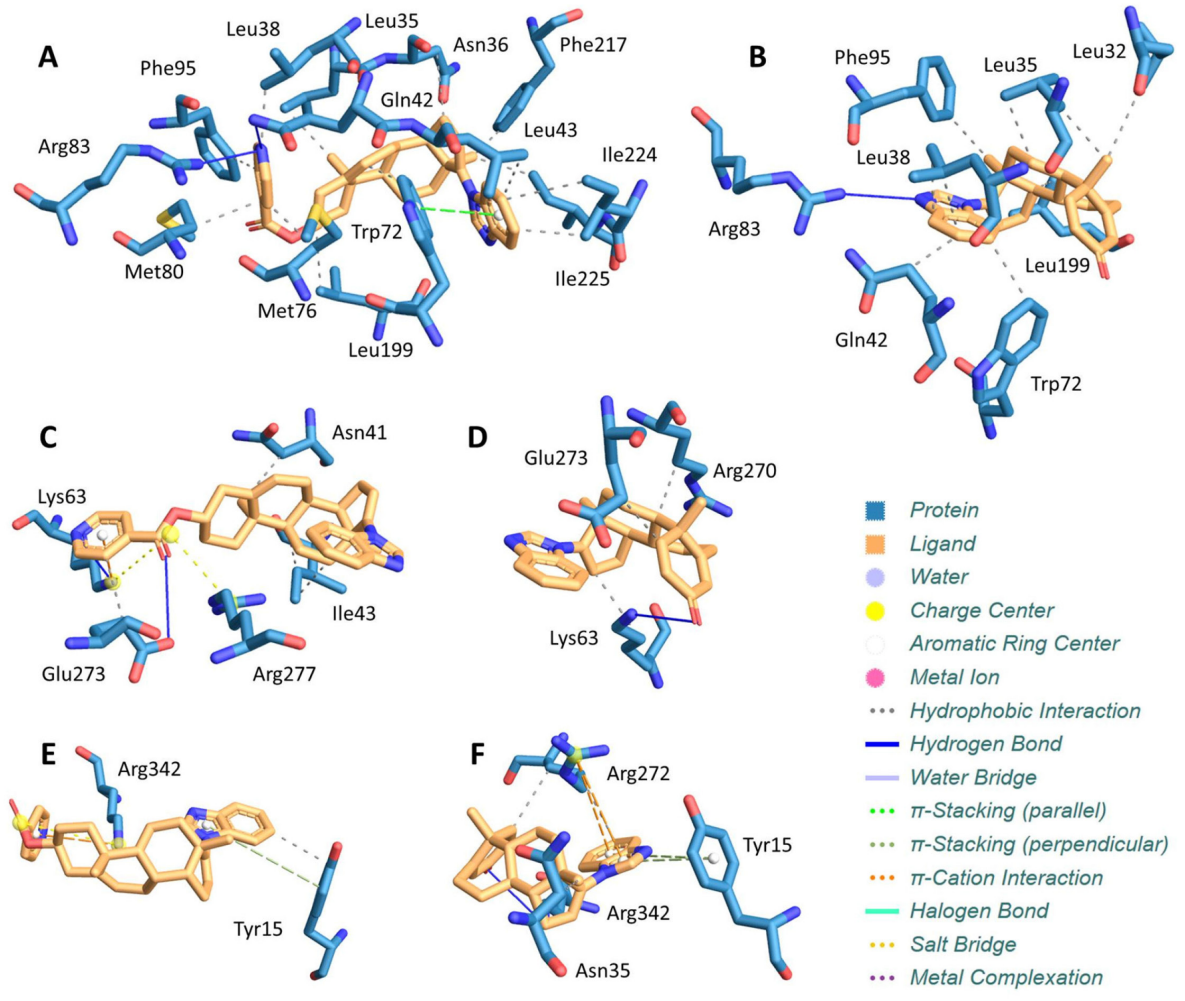
Molecular Docking of ARDAs reveals potential new targets The AR LBD and predicted interactions with VNPT-178 **(A)** and VNLG-74A **(B)** yield a net Gibb’s free energy of binding (Δ*G°*) of -7.2 and -6.6 kcal/mol, respectively. When docked to the ATPase domain of BiP/Grp78, VNPT-178 **(C)** and VNLG-74A **(D)** yield a Δ*G°* of -8.0 and -8.3 kcal/mol, respectively. Similarly, docking of VNPT-178 **(E)** and VNLG-74A **(F)** yields a Δ*G°* of -7.8 and -7.1 kcal/mol, respectively, when bound to the ATPase domain of Hsp70-1A.

Since we have demonstrated a specific capacity for VNPT-178 and VNLG-74A to antagonize the fAR, we performed molecular docking studies to estimate the binding energy of ARDA-fAR interactions. In Figures 6A-6B, VNPT-178 and VNLG-74A are docked to the ligand-binding domain of the wild-type AR based on resolved crystallographic interactions with its ligand. For a spontaneous interaction to occur, the ligand-receptor complex must be thermodynamically favored resulting in a negative change in Gibb’s free binding energy (ΔG°). Here, we predict the binding energies of VNPT-178 and VNLG-74A associated with the AR-LBD to be -7.2 and -6.6 kcal/mol, respectively. Looking next at ARDA binding to the ATPase domain of BiP/Grp78, we estimate a binding energy of -8.0 kcal/mol for VNPT-178 (Figure 6C) and -8.3 kcal/mol for VNLG-74A (Figure 6D). By comparison, based on these docking predictions both ARDAs have a potentially higher affinity for the ATPase domain of BiP/Grp78 than the AR-LBD. Inspired by these findings, we also compared VNPT-178 (Figure 6E) and VNLG-74A (Figure 6F) for binding to the ATPase domain of Hsp70-1A and again found improved binding potential versus the AR-LBD with ΔG° values of -7.8 and -7.1 kcal/mol, respectively. As these predictions align with reported ΔG° values for epigallocatechin gallate (EGCG) bound to BiP/Grp78 (-8.4 kcal/mol) and Hsp70-1A (-7.5 kcal/mol), and EGCG has been evaluated for its antiproliferative and pro-apoptotic activities in multiple cancer models, these data may create an important link between our own ARDA studies and provide novel insights for developing improved CRPC therapeutics [26, 27].

## CONCLUDING REMARKS

Expanding upon previous work with galeterone in AR-negative PC cell models, we simultaneously evaluated the activation of the UPR and induction of apoptosis relative to disruptions in AR signaling. In these studies we have identified strong evidence supporting an AR-independent activity of the ARDAs VNPT-178 and VNLG-74A capable of inducing apoptosis in LNCaP, 22Rv1, and PC-3 cells. Furthermore, based on our data we have reason to suspect that although 22Rv1 cells highly express AR-V7, they may be uniquely sensitive to the AR-independent action of these ARDAs and thus fail to accurately represent a broader context of AR-V expression in CRPC tumors.

**Figure 7:**
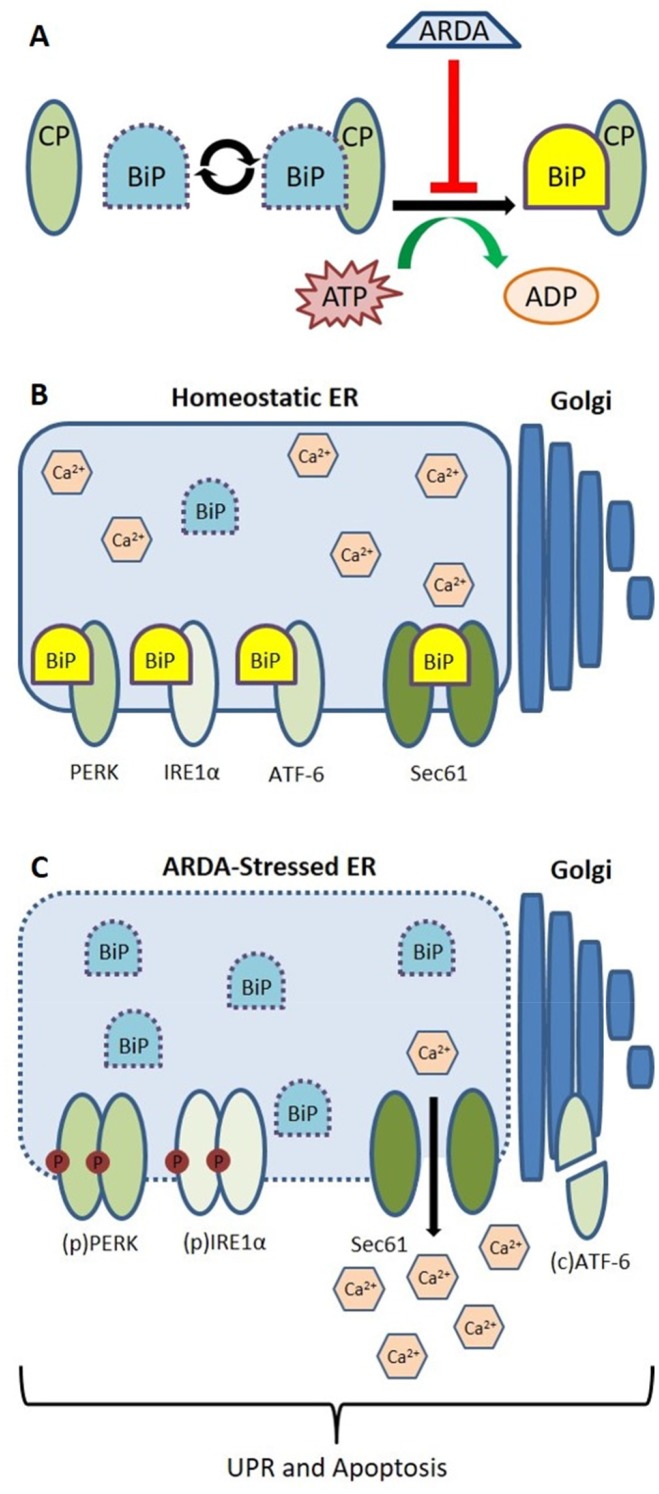
Illustrative model predicting ARDA-mediated disruption of BiP/Grp78 function and consequent induction of the UPR **(A)** When bound to ATP, BiP/Grp78 interacts transiently with its client proteins (CP). Hydrolysis of ATP to ADP drives the formation of stable (yellow) BiP•CP complexes by altering the conformation and affinity of BiP’s SBD. The ARDAs VNPT-178 and VNLG-74A bind to BiP’s ATPase domain and prevent association of stable complexes. **(B)** BiP maintains ER homeostasis through its stable interactions with ER transmembrane proteins PERK, IRE1α, ATF-6 and the Sec61 translocon. **(C)** Administration of ARDA causes a UPR and ultimately apoptosis by promoting the dissociation of BiP/Grp78 from its client proteins resulting in calcium efflux through the Sec61 translocon, the oligomerization and phosphorylation of PERK and IRE1α as well as ATF-6’s translocation to and cleavage within the Golgi.

The UPR is a powerful survival pathway allowing cells to overcome many metabolic challenges and, in solid tumors, is believed to play an important role in promoting selection of highly resilient cancer cells [28]. However, a sustained or highly active UPR can also drive apoptosis and it may be possible for otherwise broadly-acting anticancer agents to find pseudo-specificity against these tumors by pushing the UPR towards induction of cell death. Hsp70 family chaperone proteins contain two domains critical for their activity: an N-terminal ATPase domain that allosterically regulates the affinity of their C-terminal substrate-binding domain (SBD) [29]. As unfolded proteins accumulate in the ER lumen, BiP/Grp78 dissociates from PERK, IRE1α and ATF-6 to activate the UPR. Additionally, BiP/Grp78 functions as a gatekeeper for the Sec61 translocon [30] and reducing BiP/Grp78 expression or blocking its interaction with Sec61 promotes cytosolic Ca^2+^ accumulation [31]. As BiP/Grp78 expression remained stable, a direct binding of VNPT-178 or VNLG-74A may promote dissociation of BiP/Grp78 from its client proteins simultaneously activating the UPR and promoting Ca^2+^ efflux to the cytosol (Figures 7A-7C).

The AR is a putative Hsp90 client protein and depends on this interaction for the stable exposure of its hydrophobic cleft for ligand binding [32]. However, the efficient recruitment of Hsp90 to the AR requires priming by Hsp70 in an ATP-dependent process. Impeding the formation of Hsp70/Hsp90-AR complexes can simultaneously function to antagonize the fAR and promote its proteasome-mediated degradation [33, 34].

The discovery of truncated AR variants lacking a ligand-binding domain represents one of the most daunting challenges for the development of improved AR-directed therapeutics. Here, we demonstrate that while VNPT-178 and VNLG-74A are potent inhibitors of fAR signaling, their ability to induce apoptosis in LNCaP, 22Rv1, and PC-3 cells suggests an AR-independent mechanism involving activation of the UPR. This is the first study to identify specific, alternate targets of galeterone analogs and comprehensive evaluation of their activity against BiP/Grp78 and Hsp70-1A, as well as other Hsp70/Hsp90 client or ATPase domain-containing proteins, may improve our contextual understanding of the clinical utility of these novel ARDAs.

## MATERIALS AND METHODS

### Cell lines and culture

LNCaP (CRL-1740), 22Rv1 (CRL-2505), and PC-3 (CRL-1435) cells were purchased from ATCC (Manassas, VA) and cultured in Phenol red-containing RPMI-1640 media (Mediatech; Manassas, VA) supplemented with 10% standard fetal bovine serum (GE Healthcare Life Sciences; Logan, UT) and 1% penicillin:streptomycin (Gemini Bio-Products; West Sacramento, CA). All culture and treatments were performed using tissue culture-treated polystyrene dishes (Corning Inc.; Corning, NY) in humidified incubators at 37°C and 5% CO_2_. Unless otherwise indicated, media was refreshed at 24-hour intervals during treatments to maintain optimum metabolic conditions and emulate *in vivo* dynamics. For experiments running longer than 24 hours, but not multiples of 24, a media refresh schedule was customized to allow a final, 24-hour period before sample collection.

### Compounds

Galeterone, DHT, VNPT-178, VNLG-74A, and abiraterone were synthesized in-house as previously described [7, 35]. Enzalutamide (a.k.a. MDV3100, #SRP016825m) was purchased from Sequoia Research Products (Pangbourne, UK). Methyltrienolone (a.k.a. R1881, #965M935) was purchased from OChem Incorporation (Des Plaines, IL). Z-VAD-FMK (FMK001) was purchased from R&D Systems (Minneapolis, MN). Etoposide (E1383) was purchased from Sigma (St. Louis, MO). Calpeptin (SC-202516) was purchased from Santa Cruz Biotechnology (Dallas, TX). All compounds were solubilized in 95% ethanol (Pharmco-AAPER; Brookfield, CT) or ultrapure DMSO (AmericanBio; Natick, MA).

### MTT cell viability

Cells were seeded in 96-well plates at a density of 2000 (PC-3) or 3000 (LNCaP & 22Rv1) cells per well in 100 μL culture media and allowed 24 hours for attachment. The next day, compound stocks were diluted as a 2x formulation in culture media and 100 μL was added topically to each well to a final concentration within the range of 10 nM-100 μM (n=6). All plates were returned to culture incubators. After 72 hours, media was aspirated and refreshed (200 μL with indicated concentrations of each compound) and plates were returned to incubators for another 72 hours. On the final day, Thiazolyl Blue Tetrazolium Bromide (M2128; Sigma; St. Louis, MO) was solubilized in culture media as a 5x formulation and added topically to each well to a final concentration of 0.5% w/v and cells were given two hours of incubation before the media was aspirated and replaced with 100 μL DMSO. To promote cell lysis, plates were agitated on a BT1500 orbital shaker (Benchmark; Edison, NJ) for 10 minutes at 500 rpm. Absorbance was measured at 570 nm wavelength light using an ELx800 plate reader and Gen5 software (BioTek Instruments; Winooski, VT). Data was exported to and organized in Microsoft Excel; 50% and 90% Growth Inhibitory (GI) values were calculated based on a non-linear regression curve fit using GraphPad Prism4 software (La Jolla, CA). Plots represent the absorbance mean ± SEM of each [compound] versus vehicle-treated controls from at least three independent experiments.

### Colony formation

22Rv1 cells were seeded in 6-well plates at a density of 500 cells per well in 2 mL culture media (refreshed 2x weekly) containing vehicle or compound (1 or 5 μM) and adherent colony formation was evaluated on day 15 using 0.05% (w/v) Crystal Violet Staining Solution (10 mM PBS containing 1% (v/v) Formaldehyde and 1% (v/v) Methanol; pH 7.4). Images of the 6-well plates were generated using a digital scanner. Colonies were manually counted (>30 cells) and total colony area was calculated using ImageJ software with “Colony Counter” and “ColonyArea” plugins. Representative images and plots of the mean ± standard deviation of treated groups versus vehicle controls are shown. Statistical analysis was performed using Student’s *t* test and the significance level was determined by the largest *p*-value within each treatment group.

### Luciferase reporter

Competent E. *coli* (JM109) were transformed by heat-shock to express either the firefly or Renilla luciferase reporters and grown on ampicillin-containing agar plates. The firefly luciferase gene is fused to an ARR2-modified rat Probasin promoter (ARR2-Luc; kindly provided by Dr. Yun Qiu) while the Renilla luciferase reporter is pRL-null (Promega, #E2271). A single bacterial colony containing each reporter was selected and grown in 40 mL LB broth overnight (37°C, 220 rpm constant agitation). Plasmid DNA was purified using an EndoFree Plasmid Maxi Kit (Qiagen, #12362) and quantified using a BioPhotometer Plus (Eppendorf, #6132). LNCaP and 22Rv1 cells were seeded in 12-well tissue culture-treated plates and grown overnight in phenol red-free RPMI-1640 supplemented with 10% charcoal/dextran-treated FBS and 1% penicillin/streptomycin (C/D Media) to reduce androgen availability. The cells were co-transfected with the firefly and Renilla luciferase reporters using Effectene (#301425) transfection reagents in accordance with the manufacturer’s suggested protocol (Qiagen; Hilden Germany) for six hours in C/D media. After transfection, media was aspirated and replaced with C/D media containing vehicle or 10 μM of each compound for a four-hour pre-treatment before the addition of DHT (C/D media with vehicle or 10 μM compound and 20 nM DHT to be added topically 1:1 to each well to a final concentration of 10 nM). Cells were incubated for an additional 20 hours at 37°C and 5% CO_2_. Media was aspirated from each well and 200 μL of 2.5X Passive Lysis Buffer (Promega) was added. Plates were agitated on automated plate shakers for four hours at 4°C to complete cell lysis. Full-length AR transcriptional activation was analyzed using a Dual-Luciferase Reporter Assay System (E1980) in accordance with the manufacturer’s recommendations (Promega; Madison, WI). Luciferase activity was measured in white 96-well flat bottom plates (Corning) using a Tecan M1000 plate reader. For three independent experiments (n=4), mean relative luciferase units (RLU) and standard errors were calculated using Excel and presented as fold-change versus vehicle-treated positive control.

### AR/AR-V7 knockdown

Short hairpin RNAs (shRNA) specific for the human androgen receptor (AR) and AR-V7 (aka AR3) were packaged into the pLKO.1-puro vector for the transient transfection of 22Rv1 cells. The oligo sequences used for plasmid construction are as follows: fAR 5’-TGC ACT GCT ACT CTT CAG CAT TCA AGA GAT GCT GAA GAG TAG CAG TGC TTT TTT C-3’ and 5’-TC AGA AAA AAG CAC TGC TAC TCT TCA GCA TCT CTT GAA TGC TGA AGA GTA GCA GTG CA-3’; AR-V7 5’-TGT AAT AGT GGT TAC CAC TCT TCA AGA GAG AGT GGT AAC CAC TAT TAC TTT TTT TTC-3’ and 5’-TCG AGA AAA AAA AGT AAT AGT GGT TAC CAC TCT CTC TTG AAG AGT GGT AAC CAC TAT TAC A-3’.

22Rv1 cells were seeded in 6 well plates at a density of 150,000 cells per well and incubated overnight. The following day, the media was aspirated and refreshed containing an optimal dosage of mature lentivirus (empty vector, fAR shRNA, or AR-V7 shRNA). 22Rv1 cells were cultured in the presence of the lentivirus for 48 hours before the media was aspirated and refreshed with media containing vehicle or VNPT-178 for another 24 hours.

### Microscopy

Representative LNCaP, 22Rv1, and PC-3 cell images were taken at 200x total magnification using a Fisher Scientific Micromaster inverted digital microscope and the Micron software. Images shown are a 300x300 pixel crop (600x600 for vehicle controls) of the original 3MP (2048x1536) image for clarity purposes.

### Western blotting

Antibodies (primary 1:500) purchased from Santa Cruz Biotechnology (Santa Cruz, CA) include: AR N20 (SC-816, Lot I1014). Antibodies (primary 1:1000; secondary 1:3000) purchased from Cell Signaling Technology (Boston, MA) include: PSA/KLK3 (#5877, Lot 1), PARP (#9542, Lot 14), Caspase-3 (#9665, Lot 7), β-Tubulin (#2128, Lot 3), GAPDH (#2118, Lot 8), CHOP (#2895, Lot 3), LC3-A/B (#12741, Lot 3), Beclin-1 (#3495, Lot 2), PERK (#5683, Lot 5), (p) eIF2α (#3398, Lot 2), eIF2α (#5324, Lot 3), ATF-4 (#11815, Lot 4), ATF-6 (#65880, Lot 1), IRE1α (#3294, Lot 9), XBP-1s (#12872, Lot 3), Calnexin (#2679, Lot 4), BiP/Grp78 (#3177, Lot 8), ERO-1Lα (#3264, Lot 4), PDI (#3501, Lot 3), anti-Rabbit IgG (#7074, Lot 26), anti-Mouse IgG (#7076, Lot 31). Antibodies (primary 1:1000) purchased from Abcam (Cambridge, MA) include: (p) PERK (ab192591, Lot GR185975-32). Whole cell lysates were collected for each condition in RIPA buffer (#R0278; Sigma) supplemented with 0.02% EDTA (#E8008; Sigma), Complete protease inhibitor cocktail (#11697498001; Roche), and phosphatase inhibitor cocktails 1 and 2 (#P2850, #P5726; Sigma). Samples containing 15-30μg total protein were resolved by SDS-PAGE using 8, 10, or 12% polyacrylamide gels. Proteins were captured on PVDF membrane (#162-0177) purchased from Bio-Rad Laboratories (Hercules, CA) and HRP-conjugated secondary antibodies were detected using SuperSignal West Pico chemiluminescent substrate (#34080) purchased from Thermo Scientific (Rockford, IL) and X-Ray film.

### Molecular docking

The ligands VNPT-178 and VNLG-74A were docked to the AR, BiP/Grp78 and Hsp70-1A binding sites by sampling their rotational and translational degrees of freedom and the generated poses were subsequently scored by their estimated free energy of binding (Δ*G* ° ). Docking computations were performed on the AR-LBD, BiP/Grp78, and HSP70-1A crystal structures (Protein Data Bank: 2PNU, 3IUC and 5AR0, respectively). The ligand SMILES strings were first converted to 2D MOL format by Indigo (EPAM) then to 3D MOL format by OpenBabel [36]. Subsequently, the 3D MOLs were converted to PDBQT format by using a ligand preparation script of ADT [37]. The Vina docking algorithm developed by Oleg Trott et al. was used to perform the docking computations [38]. The initial binding center coordinates were derived from the sc-PDB database or original ligands co-crystallized in the structures [39]. Essential hydrogen atoms and Gasteiger charges were added. Non-polar hydrogens, lone-pairs, water molecules and non-standard residues were removed. Position, orientation, and torsions of the ligand molecule were initialized by a random seed. The binding search space was set to 22Å per dimension, and the exhaustiveness of conformational sampling was set to eight to balance search thoroughness and simulation time.

## Abbreviations

GI_50_: 50% growth inhibitory concentration
GI_90_: 90% growth inhibitory concentration
AR: androgen receptor
ARDA: androgen receptor depleting agent
AR-V: androgen receptor splice-variant
CRPC: castration-resistant prostate cancer
ER: endoplasmic reticulum
ERSR: endoplasmic reticulum stress response
fAR: full-length androgen receptor
LBD: ligand-binding domain
MOMP: mitochondrial outer membrane permeabilization
PC: prostate cancer
SBD: substrate-binding domain
UPR: unfolded protein response
